# Prognostic Interactions between FAP+ Fibroblasts and CD8a+ T Cells in Colon Cancer

**DOI:** 10.3390/cancers12113238

**Published:** 2020-11-03

**Authors:** Mercedes Herrera, Artur Mezheyeuski, Lisa Villabona, Sara Corvigno, Carina Strell, Christian Klein, Gabriele Hölzlwimmer, Bengt Glimelius, Giuseppe Masucci, Tobias Sjöblom, Arne Östman

**Affiliations:** 1 Oncology and Pathology Department, Karolinska Institutet, 17164 Stockholm, Sweden; mercedes.herrera@ki.se (M.H.); lisa.villabona@ki.se (L.V.); giuseppe.masucci@ki.se (G.M.); 2 Department of Immunology, Genetics and Pathology, Uppsala University, 75185 Uppsala, Sweden; artur.mezheyeuski@igp.uu.se (A.M.); carina.strell@ki.se (C.S.); bengt.glimelius@igp.uu.se (B.G.); tobias.sjoblom@igp.uu.se (T.S.); 3 Department of Gynecologic Oncology and Reproductive Medicine, The University of Texas MD Anderson Cancer Center, Houston, TX 77030, USA; sara.corvigno@gmail.com; 4 Roche Innovation Center Zurich, Roche Pharma Research and Early Development pRED, 8952 Schlieren, Switzerland; christian.klein.ck1@roche.com; 5 Roche Innovation Center Munich, Department of Pathology and Tissue Analytics, Roche Pharma Research and Early Development pRED, 82377 Penzberg, Germany; gabriele.hoelzlwimmer@roche.com

**Keywords:** colon cancer, tumor microenvironment, cancer associated fibroblasts, T lymphocytes, prognosis

## Abstract

**Simple Summary:**

In addition to malignant cells, tumors are composed also of other cell types including immune cells and fibroblasts. These cell types interact with each other and with the malignant cells. Prognosis associations have previously been demonstrated for CD8-positive immune cells. Recent studies suggest that fibroblasts can affect the function of immune cells. The aim of this study was to investigate if the fibroblast composition of tumors affected the prognosis association of CD8 immune cells. This study demonstrated that in colon cancer, CD8 prognosis associations was restricted to the group of tumors with high expression the FAP fibroblast marker. Our findings suggest continued mechanistic studies regarding crosstalk between FAP-positive fibroblasts and the different immune cell types; and also support the investigation of fibroblast/T-cell interactions for therapeutic purposes.

**Abstract:**

Inter-case variations in immune cell and fibroblast composition are associated with prognosis in solid tumors, including colon cancer. A series of experimental studies suggest immune-modulatory roles of marker-defined fibroblast populations, including FAP-positive fibroblasts. These studies imply that the fibroblast status of tumors might affect the prognostic significance of immune-related features. Analyses of a population-based colon cancer cohort demonstrated good prognosis associations of FAP intensity and CD8a density. Notably, a significant prognostic interaction was detected between these markers (*p* = 0.013 in nonadjusted analyses and *p* = 0.003 in analyses adjusted for cofounding factors) in a manner where the good prognosis association of CD8 density was restricted to the FAP intensity-high group. This prognostic interaction was also detected in an independent randomized trial-derived colon cancer cohort (*p* = 0.048 in nonadjusted analyses). In the CD8-high group, FAP intensity was significantly associated with a higher total tumor density of FoxP3-positive immune cells and a higher ratio of epithelial-to-stromal density of CD8a T cells. The study presents findings relevant for the ongoing efforts to improve the prognostic performance of CD8-related markers and should be followed by additional validation studies. Furthermore, findings support, in general, earlier model-derived studies implying fibroblast subsets as clinically relevant modulators of immune surveillance. Finally, the associations between FAP intensity and specific immune features suggest mechanisms of fibroblast-immune crosstalk with therapeutic potential.

## 1. Introduction

The current classification of colorectal cancer (CRC) by the AJCC (American Joint Committee on Cancer) depends on the extent of the primary tumor (T), the involvement of regional lymph nodes (N) and the presence of distant metastases (M) (TNM staging) [1]. Although powerful, TNM classification fails to provide sufficient prognostic information [2].

Immune cell infiltration has a major effect on the clinical outcome in solid tumors, and it has been associated with a favorable prognosis [3,4,5,6]. The “immunoscore”, based on the quantification of CD3- and CD8-positive lymphocyte populations, has consistently shown associations with the prognosis [7]. The “immunoscore” thus represents the true progress in CRC prognostication.

The integration of additional immune modulatory mechanisms might improve the prognostic significance of “immunoscore” or other markers related to T-cell enumeration. This notion is supported by emerging findings, including demonstrations that the prognostic capacity of an immunoscore-like metagene differing between cases with high or low expressions of checkpoint inhibitors [8].

Cancer-associated fibroblasts (CAFs) constitute a heterogeneous and functionally diverse cell population present in most solid tumors [9,10]. Early studies in mouse models identified model- and subset-specific immune-supportive or suppressive effects of different CAF subsets [11,12,13,14,15].

More recently, a series of studies have provided detailed information regarding various immune-modulatory mechanisms exerted by different marker-defined CAF subsets, including FAP-positive fibroblasts. In breast cancer, a FAP-positive CAF subpopulation promoted an immune-suppressive environment by enhancing the regulatory T-cell capacity to inhibit T-effector proliferation [16]. Melanoma-derived fibroblasts abrogate natural killer (NK) cell functions, which result in an impairment of the NK cell-mediated killing of melanoma target cells [17,18]. Moreover, CAFs drive antigen-specific immune suppression by affecting CD4-positive T cells, T-regulatory cells or CD8-positive T cells [19,20]. Other studies have also shown CAFs as regulators of responses to immune therapy [21,22].

Collectively, these studies imply that the fibroblast status of tumors might affect the prognostic significance of CD8 cells by generating microenvironments that are more or less permissive for T cell-mediated immune surveillance. Notably, this concept largely remains to be validated in clinical settings by integrated analyses of CAFs and immune features.

The fibroblast activation protein (FAP) is a cell-surface serine protease that is upregulated in CAFs in a wide variety of cancers. Thus, increasing evidence suggests that it may be expressed by some epithelial tumor cells and, also, by additional cell types of the tumor microenvironment, like macrophages, mature adipocytes and mesenchymal stromal cells [23]. Recent studies on fibroblast heterogeneity have demonstrated that FAP is not homogenously expressed on tumor fibroblasts but, rather, expressed on yet-to-be fully defined subsets of fibroblasts [16,24]. Immune-regulatory mechanisms remain to be fully identified but have been suggested to include the secretion of immune-modulatory CXCL12; the expression of OX40L, PD-L2 and JAM2 and the induction of CTLA4 and PD1 in regulatory T cells [12,16,25]. Moreover, FAP expression by stromal cells can have a profound indirect effect on the immune compartment of tumors promoting a shift in T-cell phenotypes towards T-regulatory cells [23]. Interestingly, studies on immune-regulatory mesenchymal fibroblast-like cells of lymph nodes suggest that these cells are derived from FAP+ precursor cells [26,27].

Earlier studies on prognostic associations of FAP expression have detected good prognosis as well as poor prognosis associations of FAP expression in different tumor types, including CRC [28,29,30]. Notably, none of the earlier FAP studies in CRC have addressed the possibility that FAP prognosis associations are linked to an impact of FAP-positive fibroblasts on immune surveillance.

This study investigates the prognosis associations of FAP and CD8a in two colon cancer cohorts and, specifically, explores the possibility that the prognostic capacity of CD8-positive cells differs between cases defined by their FAP status.

## 2. Materials and Methods

The paragraphs below present a description of the materials and methods used for the analyses reported in the figures and tables of the main text. For additional details and for the materials and methods for the analyses reported exclusively in the supplementary figures and tables, see the Material and Methods part of the Supplementary Materials.

### 2.1. Study Cohorts

Two independent cohorts of surgically resected colon cancers were used. For the first set of analyses, a tissue microarray (TMA) generated from a population-based CRC cohort obtained in the context of the U-CAN bio banking initiative was used (http://www.u.-can.uu.se/about-u-can/) [31]. Adjuvant treatment was composed of 5-fluorouracil alone or in combination with oxaliplatin. The reported data were derived from analyses of cores selected to represent typical areas of the tumor center. For the majority of cases, one core per case was analyzed.

The second set of analyses used tissue material derived from patients with stage II/III CRC participating in the “Nordic adjuvant randomized clinical trial” performed for the evaluation of the efficacy of 5-fluorouracil-based adjuvant chemotherapy in 1991–1997 [32].

Analyses of the two cohorts were approved by the regional ethical committees in Uppsala, Sweden (Dnr 2010/198 and Dnr 2015/419) and the ethical committee of the Karolinska Institutet, Stockholm, Sweden (Dnr 00-260, 2014/664-32), respectively.

### 2.2. Antibody Staining Procedures

FAP staining: Four-micrometer-thick sections of the TMA blocks of the U-CAN cohort, and whole-slide tumor sections from the “Nordic adjuvant randomized clinical trial”, were stained with antibodies against human Fibroblast activation protein alpha (FAP) (1:200, D8 Cat# MABS1001, Vitatex, Stony Brook, NY, USA). Antibodies were visualized using Ultramap anti rat-HRP (Horseradish Peroxidase) from Ventana Medical Systems, Inc. (Ventana, Oro Valley, AZ, USA) for antibody detection. Following antibody detection, sections were subjected to a counterstain with hematoxylin (Mayers HTX, 01820, Histolab, Askim, Sweden). For more detailed descriptions of staining procedures, see Supplementary Materials and Methods.

Single marker CD8a staining of the tumor cohort from the “Nordic adjuvant randomized clinical trial”: Whole-slide tumor sections from the “Nordic adjuvant randomized clinical trial” were stained with antibodies against human CD8a (1:100, Cat# M7103, Agilent Technologies Biotechnology, Santa Clara, CA, USA). Antibodies were visualized using an avidin-biotin-peroxidase complex (ABC) kit (Vectastain, Vector Laboratories, Burlingame, CA, USA) and chromogen 3′-diaminobenzydine system for antibody detection. Following antibody detection, sections were subjected to counterstain with hematoxylin (Mayers HTX, 01820, Histolab). For more detailed descriptions of staining procedures, see Supplementary Materials and Methods.

### 2.3. Multiplex Staining for Immune Markers

Four-micrometer-thick sections of TMA blocks of the U-CAN cohort were used for multiplex staining, as described earlier [4]. In short, the staining was performed with an Opal Multiplex IHC Kit (PerkinElmer, Waltham, MA, USA) with a panel of five antibodies: CD4, 1:200 (mouse/4B12, DAKO/M7310, Santa Clara, CA, USA), CD8a, 1:500 (mouse/144B, Thermo Fisher Scientific/MA5-13473, Waltham, MA, USA), FoxP3, 1:100 (rabbit/D6O8R, Cell Signaling Technology/12653s, Danvers, MA, USA), CD20, 1:3000 (mouse/L26, DAKO/GA604) and CD45RO, 1:200 (mouse/UCHL1, Thermo Fisher Scientific/MA1-19452) and pan-cytokeratin cocktail (E-cadherin, 1:5000 (mouse/Clone 36, BD Biosciences/610182, Franklin Lakes, NJ, USA), anti-pan cytokeratin, 1:1000 (mouse/(C-11), Abcam, San Francisco, CA, USA/ab7753) and anti-pan cytokeratin clone, 1:2000 (mouse/AE1/AE3, Thermo Fisher Scientific/MA5-13156)). 4′,6-diamidino-2-phenylindole (Spectral DAPI, PerkinElmer) was used to visualize cell nuclei.

### 2.4. FAP Scoring and Dichotomization

FAP-stained slides of the U-CAN cohort (TMA blocks) and “Nordic adjuvant randomized clinical trial” (whole-block sections) were digitalized and subsequently reviewed by two/three different evaluators. FAP intensity was scored on an optical four-point intensity scale (0 to 3). Examples are provided in Figure S1. For cases where two cores were available, average values were calculated to obtain a case value.

Exploratory analyses of associations of FAP with survival were initially done following classification of cases into three groups of as equal sizes as possible, allowed by score distributions. These three groups were subsequently reduced to two groups (“low” and “high”). The binarized FAP low/high classifications of cases were also used in the analyses of associations of FAP with clinic-pathological characteristics and in analyses of associations between markers of immune cells.

### 2.5. Manual CD8a Scoring of the “Nordic Adjuvant Randomized Clinical Trial”

CD8a semi-quantitative scoring of the stained slides of the “Nordic adjuvant randomized clinical trial” cohort was performed under light microscopy. To determine the CD8a-positive cell infiltration, the amount of positive cells in the epithelial areas of the tumor center was counted using a hotspot approach with a four-graded scale. Staining was evaluated by two investigators. In case of discordant results (more than two score levels), a third evaluator was consulted, and consensus was made.

### 2.6. Automated Scoring of Multiplex Stained Tissues

Multiplex-stained TMAs were scanned with the Vectra Polaris System (PerkinElmer) at resolution 2 pixels per 1 μm. Thereafter, spectral unmixing was performed in the Inform Software package (PerkinElmer). Image analysis procedure was described in detail previously [4]. In brief, the infiltration of immune cells was evaluated based on the marker expression level and quantified as number of positive cells per region unit. Analyses were performed separately in the stromal and tumor compartments.

### 2.7. Dichotomization of CD8a Scoring

Exploratory analyses of associations of CD8a density in tumor epithelial areas of the tumor center with survival in the U-CAN cohort was initially done following classification of cases into three groups of as equal sizes as possible allowed by score distributions. These three groups were subsequently reduced to two groups (“low” and “high”). Examples of cases classified as low or high regarding CD8a density in tumor epithelial areas of the tumor center are shown in Figure S2. For dichotomization of the CD8a density of the “Nordic adjuvant randomized clinical trial”, the 4 original scoring groups were divided to generate low/high groups with a distribution similar to the groups of the U-CAN cohort.

### 2.8. Statistical Analyses

Overall survival (OS) was defined as survival from the date of diagnosis to death due to any reason in U-CAN cohort. In the “Nordic adjuvant randomized clinical trial”, OS was the time from the date of randomization, which was about 8 weeks after surgery, to death. Marker impacts on OS were estimated using Log-rank tests. To estimate relative hazards in both univariable and multivariable models, a Cox proportional hazards model was used. For the analyses of associations between the markers and clinicopathological characteristics, the chi-square test was applied. Mann-Whitney test was used to analyze differences of the continuous immune markers between binarized marker-defined cases. To confirm statistically significant interactions between markers in the prognostication of OS, a formal interaction test was applied. The immune features ratio was calculated as the ratio of the immune cell density in the epithelial area to the immune cell density in the stroma. For those individuals that showed absence of the immune marker in the stroma area, the highest ratio observed in the studied immune cell marker was assigned. Individuals displaying absence of a specific immune marker expression in both areas were removed from the analyses.

All statistical tests were two-sided and *p*-values <0.05 considered statistically significant and were performed using SPSS V25 (SPSS Inc., Chicago, IL, USA). In case of multiple testing, Bonferroni correction was applied to adjust the critical *p*-value by using R Studio software (version 3.6.0, Rstudio, Boston, MA, USA).

## 3. Results

### 3.1. Associations between FAP Intensity and Clinico-Pathological Characteristics in the U-CAN Population-Based Colon Cancer Cohort

Analyses were performed on tissue microarray (TMA) of colon cancers from the U-CAN population composed of patients diagnosed between 2010 and 2014 in Uppsala County, Sweden. Analyses were completed for 253 cases (Figure S3). Clinicopathological characteristics of the study population are summarized in Table S1.

Case-based intensity of FAP expression in tumor stroma of the central part of the tumor were determined by two independent readers of each core using a semi-quantitative 0–3 graded scoring (Figure S1; for details regarding the evaluation of antibody specificity and dichotomization, see Materials and Methods and Supplementary Materials and Methods).

Analyses of relationships between the FAP status and clinicopathological characteristics identified a significant association between low FAP intensity and MSS (microsatellite stability) (*p* = 0.006) (Table S2). No significant associations were detected between the FAP intensity and age, gender, tumor location (right- or left-sided), stage or treatment with adjuvant chemotherapy.

### 3.2. Associations between FAP Intensity and Survival in the U-CAN Population-Based Colon Cancer Cohort

The data on FAP intensity were combined with patient survival data to explore the potential correlation between the FAP status and outcome.

**Figure 1:**
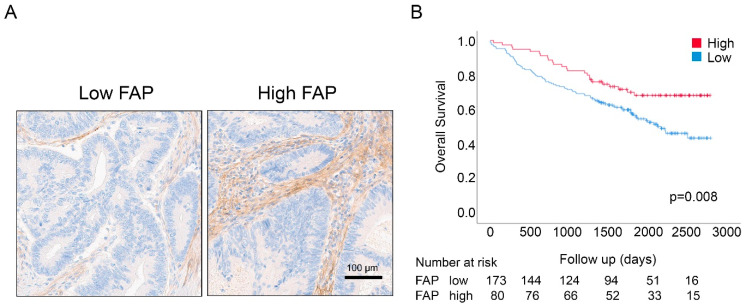
(**A**) Immunostaining of tissue microarray (TMA) cores showing low vs. high scores of FAP intensity of stromal cells in colon cancers. (**B**) Overall survival for FAP in the U-CAN cohort. Log-rank test (*p*-value) showed that patients with high FAP intensity had longer overall survival (OS) as compared to patients with low FAP intensity.

As shown in Figure 1 and Figure S4, the log-rank survival analysis demonstrated that the FAP intensity-high group displayed significantly longer OS (*p*-value = 0.008; Figure 1B). Univariable Cox regression analyses confirmed these findings and revealed a reduced risk of death for patients with high FAP intensity (Hazard Ratio (HR) = 0.54; 95% CI = 0.35–0.86; *p*-value = 0.009) (Table 1). Analyses of survival associations of other characteristics identified good prognosis associations of high CD8a density, stage I/II, age less than 66 years and adjuvant treatment (Table 1).

Multivariable Cox regression analysis, including CD8a density, age, stage, mismatch repair (MMR) status, adjuvant treatment, location and gender, demonstrated the independent prognostic significance of FAP status in this cohort (HR = 0.54; 95% CI = 0.34–0.87; *p*-value = 0.011) (Table 1).

Exploratory subset analyses were performed to explore if the prognostic significance of FAP intensity was particularly strong in patient groups defined by age, stage, MMR status, adjuvant treatment and location. According to these analyses, the FAP intensity association with survival was particularly strong in the MSI (microsatellite instability) group, in stage I/II patients, in non-adjuvant-treated and among individuals older than 66 years (Figure S5).

Two other fibroblast-related markers, PDGFRβ intensity and stroma abundance, were also analyzed with regard to prognostic capacity. None of these markers showed significant survival associations in this cohort (Figure S6).

### 3.3. Prognostic Interactions between FAP Intensity and CD8a Density in the U-CAN Population-Based Colon Cancer Cohort

The tumor cohort described above was subjected to analyses regarding CD8a density in tumor epithelial areas of the central parts of the tumor (Figure S2; see Materials and Methods for details).

Initial analyses demonstrated that a high CD8a density was significantly associated with the presence of MSI (microsatellite instability) (*p* = 0.001) and advanced stages (*p* = 0.017) (Table S3). However, no associations were found with age, gender, location and adjuvant treatment (Table S3) or FAP status (Table S2).

Moreover, a high CD8a density was associated with longer survival in univariable analyses (Table 1 and Figures S2 and S7). This significant association between CD8a status and OS was not detected in multivariable Cox regression analyses adjusted for FAP intensity, age, stage, MMR status, adjuvant treatment, location and gender (Table 1).

**Figure 2:**
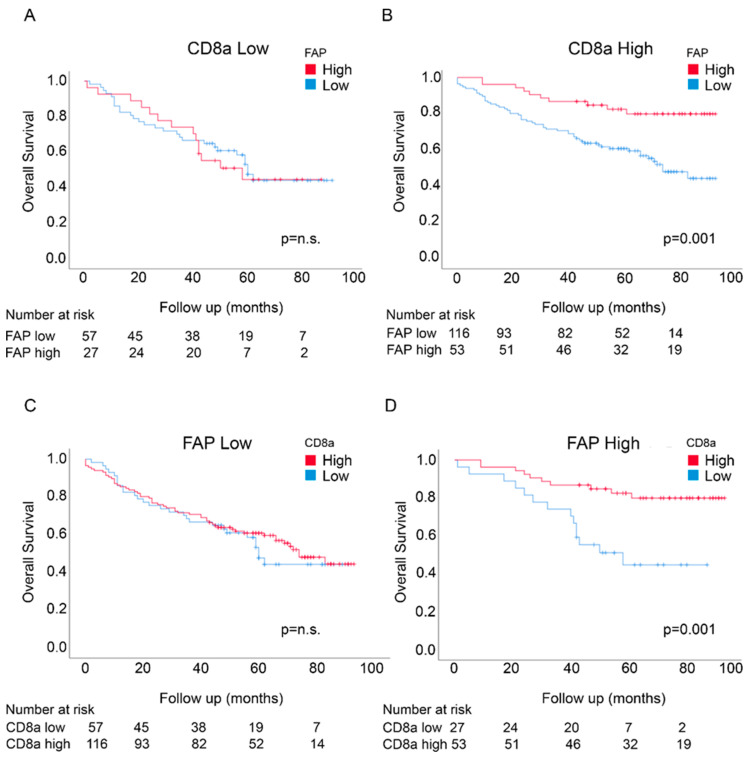
Overall survival in the U-CAN cohort for FAP intensity in patients expressing low levels of CD8a density (**A**) and high levels of CD8a density (**B**) and for CD8a density in patients expressing low levels of FAP intensity (**C**) and high levels of FAP intensity (**D**).

Potential prognostic interactions between FAP intensity and CD8a density were initially analyzed by investigating the prognostic significance of FAP intensity in CD8a-defined subgroups and of CD8a density in FAP-defined subgroups. Notably, the significant survival associations of FAP intensity and CD8a density, as determined by Log-rank tests, were restricted to the high FAP intensity in the CD8a density-high group and high CD8a density in the FAP intensity-high group, respectively (Figure 2).

A formal interaction test was employed to further analyze the data and demonstrated a significant prognostic interaction between FAP intensity and CD8a density in nonadjusted analyses (*p* = 0.013). Statistically significant interactions were also detected in adjusted analyses, including age, stage, MMR status, adjuvant treatment, location and gender (*p* = 0.003) (Table S4).

Furthermore, multivariable Cox regression analyses identified FAP intensity as an independent marker of prognosis in the CD8a density-high group (*p* < 0.001; Table S5A) and CD8a density as an independent marker of prognosis in the FAP intensity-high group (*p* = 0.003; Table S5B).

Exploratory subset analyses were performed to investigate if the prognostic interactions were particularly strong in some patient subsets. As shown in Figure S9 and Table S6, some differences were noted when cases were analyzed separately based on gender, MRR status, location or stage. The prognostic interactions were most clearly observed in males, MSS cases, “right location” cases and stage III/IV cases.

### 3.4. Prognostic Interactions between FAP Intensity and Stromal CD8a Density in an Independent Randomized Trial-Derived Colon Cancer Cohort

For consolidation of the findings from the U-CAN cohort, analyses were performed on a colon cancer tissue cohort from the “Nordic adjuvant randomized clinical trial”, investigating benefits of adjuvant 5-fluorouracil (5-FU)-based chemotherapy, which have been extensively used for biomarker studies [32,33,34].

A consort diagram of the analyses is shown in Figure S8, and clinicopathological characteristics for the population are summarized in Table S1. Analyses were completed for 267 cases.

Cases were classified according to FAP intensity and CD8a density (see Materials and Methods for details). No significant associations were detected between FAP intensity and age, gender, tumor location, stage, tumor differentiation and treatment with adjuvant chemotherapy, whereas a high CD8a density showed associations with a poor differentiation status (Table S7).

**Figure 3:**
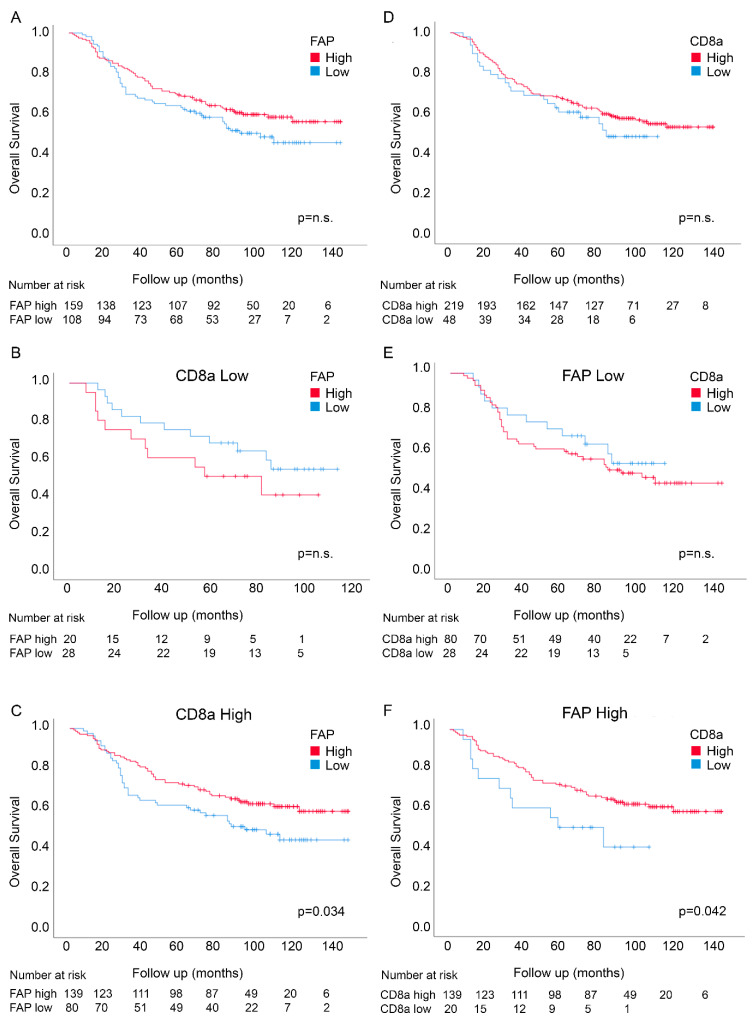
Overall survival according to FAP intensity in fibroblasts (**A**) and CD8a density (**D**) in the tumor center in the Nordic adjuvant trial. Overall survival for FAP intensity in patients expressing low levels of CD8a density (**B**) and high levels of CD8a density (**C**) and for CD8a density in patients expressing low levels of FAP intensity (**E**) and high levels of FAP intensity (**F**).

Neither FAP intensity nor CD8a density showed significant associations with survival in univariable analyses, although both markers showed trends consistent with the findings from the U-CAN cohort (Table 2 and Figure 3). FAP intensity, but not CD8a density, acted as an independent prognostic marker in multivariable analyses also, including CD8a density, age, stage, MMR status, adjuvant treatment, location, tumor differentiation and gender (Table 2).

Analyses of the prognosis associations of FAP intensity in CD8a density-defined subgroups and of CD8a density in FAP intensity-defined subgroups showed similar results as in the U-CAN cohort, with significantly good prognosis associations of FAP intensity in the CD8a density-high group (Figure 3; *p* = 0.034; Log-rank test) and of CD8a density in the FAP intensity high-group (Figure 3; *p* = 0.042; Log-rank test). As in the U-CAN cohort, statistically significant interactions were detected between the two markers using nonadjusted analyses (*p* = 0.048) (Table S8). A trend for interactions was observed in adjusted analyses also, including, age, stage, MMR status, adjuvant treatment, location, differentiation and gender (*p* = 0.093) (Table S8).

### 3.5. Associations between FAP Intensity and Immune Features

The finding of the significant prognostic impact of high FAP intensity in CD8a density-high groups in both cohorts (Figure 2B and Figure 3C) prompted efforts to identify the immune features differing between tumors with high vs. low FAP intensity in the subgroup of CD8a density-high patients. Data on the total tumor density, density in the tumor stroma and density in tumor epithelial areas of cells positive for CD4, FoxP3 and CD45RO were therefore collected from the CD8a-high cases of the U-CAN study population (see Materials and Methods for details; Figure S10).

**Figure 4:**
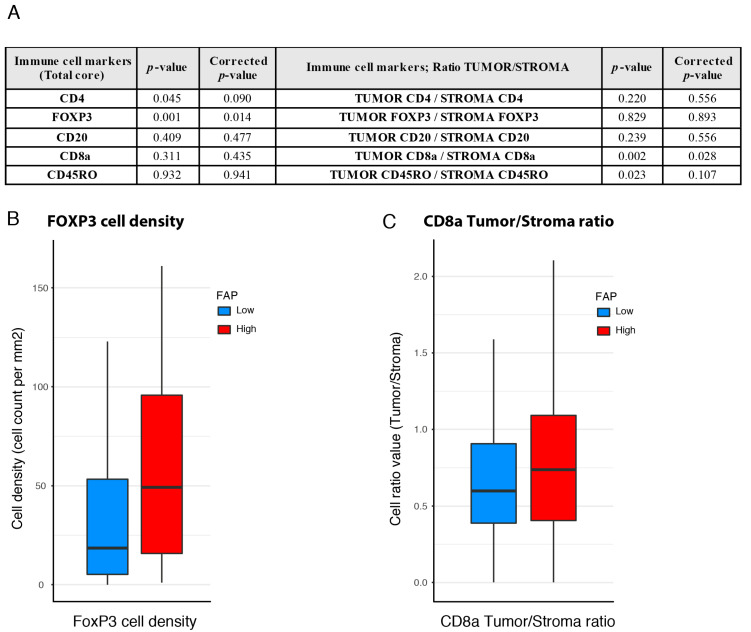
Immune characteristics of FAP status-defined subgroups of CD8a density-high cases in the U-CAN population. (**A**) Total tumor densities and ratio of the epithelial-to-stromal density of immune markers in the FAP intensity-defined cases. (**B**) FoxP3 density in the FAP intensity-defined groups within the CD8a density-high cases. (**C**) The ratio of epithelial-to-stromal density of CD8a density cells in the FAP-defined groups within the CD8a density-high cases.

A significantly higher density of FoxP3-positive immune cells was detected in the FAP intensity-high group as compared to FAP intensity-low (Figure 4A,B). Furthermore, the FAP intensity-high group also displayed a higher ratio of epithelial-to-stromal density of CD8a cells (Figure 4C).

## 4. Discussion

**Figure 5:**
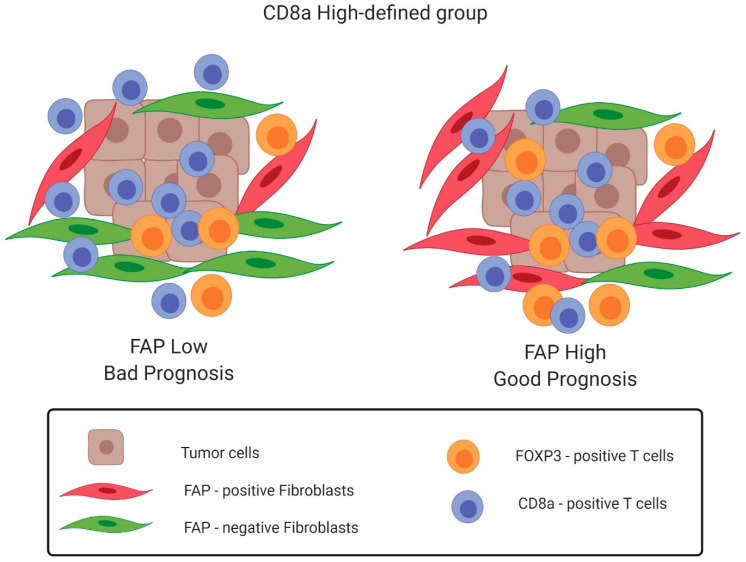
Schematic representation of the findings of this study. FAP-defined subgroups of CD8a density-high colon cancers display differences in the composition of the immune environment and differ significantly with regard to outcome. The FAP intensity-low subset shows a lower density of FoxP3-positive immune cells, and a lower ratio of epithelial-to-stromal density of CD8a cells presents a bad prognosis, while the FAP intensity-high subset displays significantly a higher density of FoxP3-positive immune cells; a higher ratio of epithelial-to-stromal density of CD8a cells presents a good prognosis. This image was created with Biorender.com.

This study identifies two FAP-defined subgroups of CD8a density-high colon cancers, which differ significantly with regard to outcome and, also, display differences in the composition of the immune environment (Figure 5). Furthermore, it is demonstrated that CD8a density lacks prognostic significance in the FAP intensity-low subset of colon cancer.

The findings are novel and relevant both from a biomarker and a tumor biology perspective. From a biomarker perspective, the study suggests possibilities to improve the prognostic performance of CD8-related markers such as the “immunoscore” [7,35]. For this purpose, additional validation studies are needed. These studies should also allow further validation of the preliminary indications that the prognostic interactions are particularly prominent in certain subsets of patients, such as males, and cases with MSS tumors. Technical aspects to consider in future studies include stringent and standardized scoring criteria for both CD8a and FAP. Furthermore, focused analyses should be done to explore if the survival interactions reflect the effects of CD8a and FAP on the intrinsic aggressiveness of the disease, on the response to adjuvant treatment or both. According to the exploratory subset analyses of Table S6, the significant interactions between markers was detected in cases not receiving adjuvant therapy, suggesting that the underlying biology is related to the intrinsic aggressiveness of the disease.

These future studies should also investigate the potential overlap between the FAP intensity-low group, not showing any prognostic association with CD8a density, and earlier proposed CD8-independent subgroups, such as cases expressing high levels of checkpoint inhibitors [8].

Regarding tumor biology, findings of the present study provide novel support, based on analyses of clinical samples, for emerging preclinical data, implying important immune-regulatory effects of fibroblast subsets [36]. One overall hypothesis that can guide future studies is that a high FAP expression, in CD8-high tumors, allows CD8 cells to exert their antitumoral effects, whereas CD8 cells in CD8a-high tumors with low FAP expression remain inactive.

The comparisons of immune features between the prognostically distinct FAP intensity-defined groups of CD8a density-high cases identified some differences that might be mechanistically linked to the good prognosis of this group. Furthermore, future studies should include an in vitro analysis and simultaneous staining of FAP and CD8a on colon human tissue samples to explore potential functional crosstalk between FAP-positive fibroblasts and CD8a-positive T cells.

The good prognosis CD8a density-high/FAP intensity-high cases showed a significantly higher density of FoxP3-positive T cells (Figure 4A). FoxP3 density has been associated with good prognosis in CRC in earlier studies [37,38,39,40,41]. The mechanisms underlying the good prognosis associations of FoxP3 T cells in CRC remain to be conclusively defined but have been suggested to include the attenuation of progression-driven inflammation. Previous studies indicated that gastrointestinal bacteria trigger cascades of proinflammatory cytokines, which can exert tumor-enhancing effects [42]. In this context, the accumulation of FoxP3 cells has been considered a favorable process. Our findings are compatible with a model where the recruitment of such inflammation-attenuating and progression-protective FoxP3-positive T cells is controlled by FAP-positive fibroblasts. These notions obviously require further functional studies. Preliminary support is available from demonstrations that FAP-positive S1 breast cancer fibroblasts promote the formation of FoxP3-positive T cells in a manner involving B7H3, CD73 and DPP4 [16].

Another immune feature of the good prognosis-associated FAP intensity-high subset is an increased epithelial/stroma ratio of CD8a density (Figure 4). Fibroblast-regulated differences in the compartmentalization of T-cells, or other immune cells, have not been well-studied in human cases of colon cancer or other tumor types. The findings of the present study suggest additional studies in other tumor types exploring the possibility that fibroblasts affect the intra-tumoral migration of extravasated immune cells. Some preclinical findings have been made that can guide such further studies. Lymph node biology studies suggest important roles for FAP-positive mesenchymal cells in controlling the migration and compartmentalization of T cells [26]. Studies in mouse cancer models have also shown that the extracellular matrix make-up, controlled by fibroblasts, can affect intra-tumoral immune cell migration [43]. Additionally, the chemokine-dependent control of T-cell retention in lymph nodes by fibroblast reticular cells has been demonstrated [44].

In this study, a high FAP intensity analyzed as a single marker was identified as an independent marker of good prognosis in both study cohorts (Figure 2 and Figure 3 and Table 1 and Table 2). The exploratory subset studies (Figure S5) indicated particularly strong prognostic associations of FAP in older cases and stage I/II cases. The mechanisms underlying the differential prognosis associations of FAP in different subsets remain unknown, and the findings should be further validated and analyzed. However, it is noted that stage I/II tumors show higher CD8 density (Table S3).

Earlier studies on the relationships between FAP status and survival have yielded conflicting results with the detection of either good or bad prognosis associations [28,29,30]. A series of differences between studies can be noted. A key issue is differences in study populations that, as indicated by the subset analyses of Figure S5, could affect the results. Technical issues such as the antibody used for FAP detection and scoring systems could also contribute to differences in the study results. In this context, it should be noted that the present findings were obtained through analyses of two independent cohorts and using an antibody that underwent stringent evaluations with regard to specificity [45]. Furthermore, the case-based status was determined following the scoring of two independent evaluators, and the scoring of both tissue cohorts was consistently done on central parts of the tumor.

## 5. Conclusions

In conclusion, novel potentially clinically relevant prognostic interactions were detected between FAP intensity and CD8a density. The findings merit continued studies and validation in independent colon cancer cohorts towards the development of novel biomarkers of clinical utility. Additionally, the findings suggest continued mechanistic studies regarding crosstalk between FAP+ fibroblasts and different immune cell types. Importantly, the study also supports emerging concepts of fibroblasts as clinically relevant modulators of immune surveillance and suggest fibroblast/T-cell interactions that can be exploited for therapeutic purposes following a better understanding of the underlying molecular processes.

## Figures and Tables

**Table 1:** Univariable and multivariable Cox models of FAP intensity and CD8a density predicting the overall survival of colon cancer patients in the U-CAN cohort.

Cox Regression Analysis	Univariable Cox Model				Multivariable Cox Model			
Covariates	HR	95.0% CI for HR		*p*-Value	HR	95.0% CI for HR		*p*-Value
		Lower	Upper			Lower	Upper	
FAP intensity TC (high vs. low)	0.545	0.346	0.859	0.009 **	0.539	0.335	0.868	0.011 *
CD8a density TC (high vs. low)	0.651	0.440	0.963	0.032 *	0.694	0.452	1.066	0.095
Age (≥66 years or <66)	1.786	1.116	2.856	0.016 *	1.243	0.728	2.122	0.425
Stage (III_IV vs. I_II)	2.941	1.905	4.540	0.000 ***	6.425	3.915	10.545	0.000 ***
MMR status (MSS vs. MSI)	1.405	0.835	2.365	0.200	1.139	0.640	2.027	0.659
Adjuvant treatment (Yes vs. No)	0.515	0.332	0.797	0.003 **	0.220	0.129	0.375	0.000 ***
Location (Right or Left)	1.075	0.734	1.575	0.709	1.202	0.790	1.829	0.391
Gender (male vs. female)	1.053	0.719	1.541	0.791	0.738	0.489	1.115	0.149

* *p* < 0.05, ** *p* < 0.01, *** *p* < 0.001; HR (Hazard Ratio); TC (Tumor Center); MMR (Mismatch repair).

**Table 2:** Univariable and multivariable Cox models of FAP intensity and CD8a density predicting the overall survival of colon cancer patients in the “Nordic adjuvant randomized clinical trial”.

Cox Regression Analysis	Univariable Cox Model				Multivariable Cox Model			
Covariates	HR	95.0% CI for HR		*p*-Value	HR	95.0% CI for HR		*p*-Value
		Lower	Upper			Lower	Upper	
FAP intensity TC (high vs. low)	0.753	0.524	1.080	0.123	0.629	0.415	0.952	0.029 *
CD8a density TC (high vs. low)	0.794	0.503	1.254	0.323	1.050	0.604	1.824	0.863
Age (≥66 years or <66)	1.280	0.882	1.857	0.194	1.323	0.877	1.995	0.182
Stage (III_IV vs. I_II)	2.067	1.421	3.008	0.000 ***	2.242	1.478	3.402	0.000 ***
MMR status (MSS vs. MSI)	2.190	1.173	4.088	0.014 *	2.294	1.182	4.455	0.014 *
Adjuvant treatment (Yes vs. No)	1.075	0.750	1.540	0.694	0.980	0.660	1.455	0.920
Location (Right or Left)	0.828	0.578	1.186	0.302	1.199	0.795	1.808	0.388
Differentiation grade (Low vs. High)	1.201	0.780	1.849	0.406	1.244	0.752	2.056	0.395
Gender (male vs. female)	1.188	0.826	1.709	0.352	1.273	0.849	1.911	0.243

* *p* < 0.05, *** *p* < 0.001; HR (Hazard Ratio); TC (Tumor Center); MMR (Mismatch repair).

## Supplementary Materials

The following are available online at https://www.mdpi.com/2072-6694/12/11/3238/s1: Figure S1. FAP intensity optical scale score in the stroma of colon cancer tissues: 0 = no staining; 1 = low; 2 = medium; 3 = high. (Brown = FAP; Blue = Hematoxylin nuclear staining). Figure S2. CD8a infiltration in colon cancers. Multiplex immunofluorescence of TMA cores showing low vs high CD8a density in tissue sections of colon cancers. CD8a+ T cells in red; cell nuclei in dark blue/DAPI and cancer cells (cytokeratin) in cyan. Figure S3. Consort flowchart showing patients from the U-CAN cohort included in the present biomarker study. TC = tumor center; IM = invasive margin. Figure S4. Overall survival curves for low, medium and high stromal FAP intensity in the tumor center in colon cancer patients from the UCAN cohort. Log-rank test (*p*-value) showed that patients with high FAP intensity had longer overall survival as compare patients with low FAP intensity. Figure S5. Forrest plot of FAP intensity in clinic-pathological subgroups. The relative impact on overall survival, displayed as hazard ratio (HR) of the FAP intensity differs according to the clinic-pathological subgroups. Figure S6. Overall survival curves for low, medium and high stromal PDGFRβ intensity and stromal fraction in colon cancer patients from the U-CAN cohort. (A) Kaplan-Meier graph shows that there are no significant differences in overall survival in patients presenting different PDGFRβ intensity in the tumor center. (B) Kaplan-Meier graph shows that there are no significant differences in OS in patients displaying different stroma fraction in the tumor center. Figure S7. Overall survival curves for low, medium and high CD8a density in the tumor center in colon cancer patients from the U-CAN cohort. (C) Log-rank test (*p*-value) showed that patients with high CD8a density had longer overall survival as compare patients with low CD8a density. Figure S8. Consort flowchart showing patients from the “Nordic adjuvant randomized clinical trial” cohort included in the present biomarker study. TC = tumor center; IM = invasive margin. Figure S9. Overall survival in the U-CAN cohort for CD8 density in subsets of patients expressing low or high of FAP intensity (A) Overall survival for CD8 density-defined cases in male and female patients with low or high of FAP intensity (B) Overall survival for CD8 density-defined cases in patients with “left-“or right-sided” tumors with low or high of FAP intensity (C) Overall survival for CD8 density-defined cases in patients with stage I/II or stage III/IV with low or high of FAP intensity. Figure S10. Multiplex immunofluorescence of a TMA core of CD8a and FOXP3 positive immune cells in a colon cancer from the U-CAN cohort. CD8a positive T cells in green; FOXP3 positive T cells in red; cell nuclei (DAPI) in dark blue and cancer cells (cytokeratin) in pink. Table S1. Clinico-pathologic characteristics of colon cancer cohorts. Table S2. Clinico-pathological characteristics of patients in U-CAN cohort and their association with FAP intensity in the tumor center. Table S3. Clinico-pathological characteristics of the patients in the U-CAN cohort and their association with CD8a density in the tumor center. Table S4. Formal interaction test showing statistically significant interactions between FAP intensity and CD8a density markers in the prognostication of OS in the U-CAN cohort. Table S5. (A) Multivariable models of FAP intensity predicting survival of colon patients with low and high expression of CD8a in the U-CAN cohort. (B) Multivariable models of CD8a density predicting survival of colon patients with low and high expression of FAP in the U-CAN cohort. Table S6. Formal interaction test showing statistically significant interactions between FAP intensity and CD8a density markers in the prognostication of OS in subsets of patients in the U-CAN cohort. Table S7. Clinico-pathological characteristics of patients in the “Nordic adjuvant randomized clinical trial” and their association with FAP intensity and CD8a density in the tumor center. Table S8. Formal interaction test showing statistically significant interactions between FAP intensity and CD8a density markers in the prognostication of OS in the “Nordic adjuvant randomized clinical trial”.
