# Supplementary material for: Prognostic Interactions between FAP+ Fibroblasts and CD8a+ T Cells in Colon Cancer
Source: Cancers (Basel). 2020 Nov 3;12(11):3238. doi: 10.3390/cancers12113238 (PMC7693786; doi:10.3390/cancers12113238)
Supplement: Supplementary file 1 [file cancers-12-03238-s001.zip › cancers-854260-suppl.-final/Supp Figures/Supplementary Figure Legends.docx]

**Supplementary Figure Legends**

**Figure S1.** FAP intensity optical scale score in the stroma of colon cancer tissues: 0= no staining; 1= low; 2= medium; 3= high. (Brown=FAP; Blue=Hematoxylin nuclear staining).

**Figure S2.** CD8a infiltration in colon cancers. Multiplex immunofluorescence of TMA cores showing low vs high CD8a density in tissue sections of colon cancers. CD8a+ T cells in red; cell nuclei in dark blue/DAPI and cancer cells (cytokeratin) in cyan.

**Figure S3.** Consort flowchart showing patients from the U-CAN cohort included in the present biomarker study. TC= tumor center; IM = invasive margin

**Figure S4.** Overall survival curves for low, medium and high stromal FAP intensity in the tumor center in colon cancer patients from the UCAN cohort. Log-rank test (p-value) showed that patients with high FAP intensity had longer overall survival as compare patients with low FAP intensity.

**Figure S5.** Forrest plot of FAP intensity in clinic-pathological subgroups. The relative impact on overall survival, displayed as hazard ratio (HR) of the FAP intensity differs according to the clinic-pathological subgroups.

**Figure S6.** Overall survival curves for low, medium and high stromal PDGFRβ intensity and stromal fraction in colon cancer patients from the U-CAN cohort. **A)** Kaplan-Meier graph shows that there are no significant differences in overall survival in patients presenting different PDGFRβ intensity in the tumor center. **B)** Kaplan-Meier graph shows that there are no significant differences in OS in patients displaying different stroma fraction in the tumor center.

**Figure S7.** Overall survival curves for low, medium and high CD8a density in the tumor center in colon cancer patients from the U-CAN cohort. **C)** Log-rank test (p-value) showed that patients with high CD8a density had longer overall survival as compare patients with low CD8a density.

**Figure S8.** Consort flowchart showing patients from the “Nordic adjuvant randomized clinical trial” cohort included in the present biomarker study. TC= tumor center; IM = invasive margin

**Figure S9.** Overall survival in the U-CAN cohort for CD8 density in subsets of patients expressing low or high of FAP intensity **A)** Overall survival for CD8 density-defined cases in male and female patients with low or high of FAP intensity **B)** Overall survival for CD8 density-defined cases in patients with “left-“or right-sided” tumors with low or high of FAP intensity **C)** Overall survival for CD8 density-defined cases in patients with stage I/II or stage III/IV with low or high of FAP intensity.

**Figure S10.** Multiplex immunofluorescence of a TMA core of CD8a and FOXP3 positive immune cells in a colon cancer from the U-CAN cohort. CD8a positive T cells in green; FOXP3 positive T cells in red; cell nuclei (DAPI) in dark blue and cancer cells (cytokeratin) in pink.
