# Supplementary material for: Prognostic Interactions between FAP+ Fibroblasts and CD8a+ T Cells in Colon Cancer
Source: Cancers (Basel). 2020 Nov 3;12(11):3238. doi: 10.3390/cancers12113238 (PMC7693786; doi:10.3390/cancers12113238)
Supplement: Supplementary file 1 [file cancers-12-03238-s001.zip › cancers-854260-suppl.-final/Supp Materials and Methods.docx]

**Supplementary Material and Methods**

**Immunohistochemistry procedures**

*FAP staining* was performed following a VENTANA-ROCHE protocol and reagents using the automated staining instrument Ventana Benchmark XT. After the de-paraffinization, slides underwent cell conditioning with Conditioner #1, Mild CC1, and Standard CC1. Then they were stained with the monoclonal rat antibody clone D8 (Vitatex, Stony Brook, NY, USA; cat. # MABS1001) at a 1:200 dilution in antibody diluent (Dako, Copenhagen, Denmark; cat. # S3022) as primary antibody and with the UltraMap anti Rat-HRP secondary antibody, and finally visualized using the Ultraview DAB detection system (Ventana, Tucson, AZ, USA; cat. #760-4456). After that tissues were counterstained manually with hematoxylin (Mayers HTX, 01820, Histolab, Sweden) before mounting.

FAP antibody specificity was confirmed regarding tissue and cell compartment localization. Positive and negative controls (colorectal cancers) were processed identically. For negative controls, the primary antibody was replaced by non-immune serum of the same species. FAP (clone D8) IHC staining reproducibility and stability was confirmed using serial sections of 3 different tumor samples in a run-to-run-, and slide-to-slide-comparing validation study. Slide aging testing was additionally performed and showed reproducible results for aging slides up to 3 weeks.

*CD8a single staining* of the tumor collection from the “Nordic adjuvant randomized clinical trial” was performed on 4 µm tissue sections that were de-paraffined, re-hydrated and rinsed with water. The samples were heated in a microwave for 20 min for antigen retrieval. The slides were left for 30 min in 0.5% H_2_O_2_ in water, finally rinsed in water and twice for 5 min in tris-buffered saline (TBS). Blocking was performed with 1% bovine serum albumin (BSA) in TBS in a moist chamber for 30 min before the sections were stained with the primary antibody at +8°C overnight. Avidin–biotin–peroxidase complex (ABC) kit (Vectastain, Vector Laboratories) was used for antigen detection. The sections were rinsed three times for 10 min each in TBS followed by incubation with the secondary antibody for 40 min. After three washing steps for three min in TBS, an ABC reagent was added for 40 min before the slides were again rinsed three times for 10 min in TBS. The immunolabelling was developed with the chromogen 3′-diaminobenzydine (15 mg/50 ml TBS for 6 min), and hematoxylin (Mayers HTX, 01820, Histolab, Sweden) was applied as a counter stain.

*PDGFR β staining* was done on four µm thick tissue sections. Sections were de-paraffinized and rehydrated. Following antigen retrieval was performed by boiling during 5 minutes in pH 10.0 buffer in decloaking chamber (Biocare Medical) at 110 degrees C. After incubation with blocking solution for 30 min sections were incubated overnight with PDGFR-β rabbit monoclonal antibody (1:100, #3169, Cell Signaling Technology, Danvers, MA). Signal amplification was performed with the polymer system (ImmPRESS™-AP Polymer Anti-Rabbit IgG MP-5401) by incubation during one hour at room temperature and visualized with Vector^®^ Blue AP Substrate Kit (SK-5300, Vector Laboratories, Burlingame, CA). The procedure is described in details before [1].

**Automated scoring of PDGFR β stained tissues**

Scoring of stromal PDGFR β expression was done with digital-image-analyses as described earlier [2]. Examples of cases classified as low, medium or high regarding PDGFR β expression in the stroma areas of the tumor center are provided in Figure S1 of Supp. M&M.

**Manual scoring of the stroma fraction**

Digitalized stained slides of the U-CAN cohort (TMA blocks) were reviewed by two different evaluators. The stroma fraction was scored by two different evaluators independently on a 5-point scale (0: 0% stroma area; 1: 1-10%; 2: 11-50%; 3: 51-75%; 4: 76-100%). For cases where two cores were available, average values of the stroma fraction were calculated to obtain a case value. Examples are shown in Figure S2 of Supp. M&M**.**


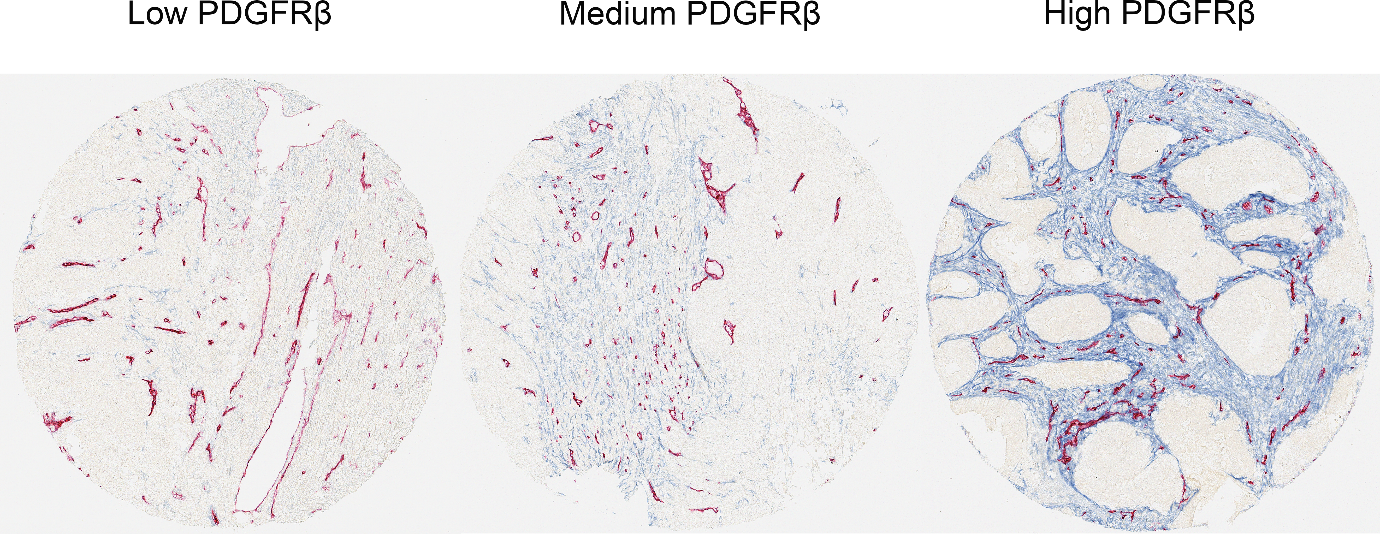


**Figure S1 of Supp. M&M.** Representative examples of colon cancer tissues with low, medium and high PDGFR β intensity in the stroma (Blue= PDGFR β; Red=CD34).


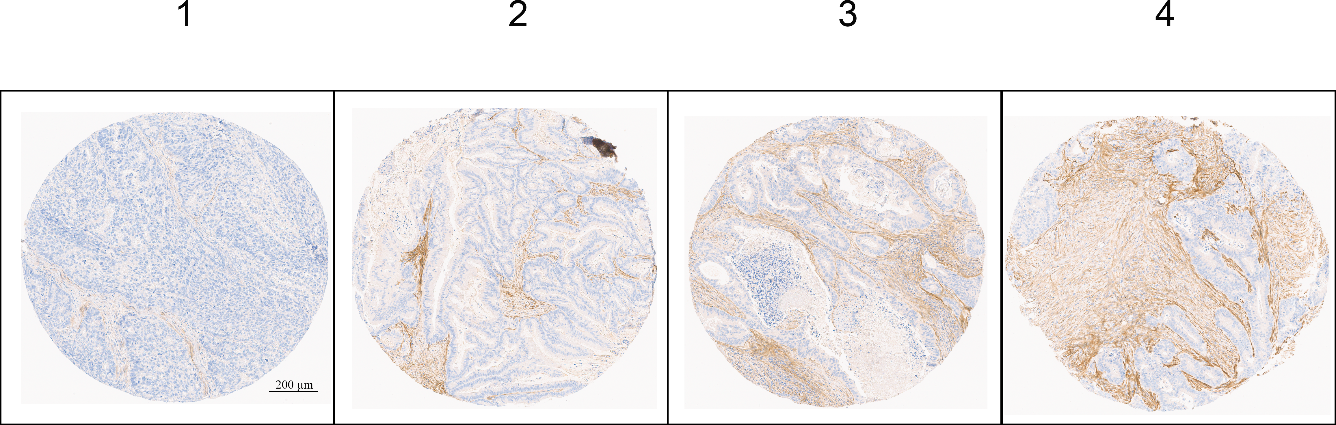


**Figure S2 of Supp. M&M.** Representative colon cancer tissues examples of cases with different stroma fraction score; 1= 1-10%; 2= 11-50%; 3= 51-75%.; 4=76-100% presence of stroma cells in relation to the total core area (Brown=FAP; Blue=Hematoxylin).

**References**

1. Mezheyeuski, A.; Hrynchyk, I.; Herrera, M.; Karlberg, M.; Osterman, E.; Ragnhammar, P.; Edler, D.; Portyanko, A.; Ponten, F.; Sjöblom, T.; et al. Stroma-normalised vessel density predicts benefit from adjuvant fluorouracil-based chemotherapy in patients with stage II/III colon cancer. *Br. J. Cancer* **2019**, *121*, 303–311.

2. Corvigno, S.; Wisman, G.B.A.; Mezheyeuski, A.; van der Zee, A.G.J.; Nijman, H.W.; Åvall-Lundqvist, E.; Östman, A.; Dahlstrand, H. Markers of fibroblast-rich tumor stroma and perivascular cells in serous ovarian cancer: Inter- and intra-patient heterogeneity and impact on survival. *Oncotarget* **2016**, *7*, 18573–18584.
