# Supplementary material for: Prognostic Interactions between FAP+ Fibroblasts and CD8a+ T Cells in Colon Cancer
Source: Cancers (Basel). 2020 Nov 3;12(11):3238. doi: 10.3390/cancers12113238 (PMC7693786; doi:10.3390/cancers12113238)
Supplement: Supplementary file 1 [file cancers-12-03238-s001.zip › cancers-854260-suppl.-final/Supp Tables/Table S1.docx]

**Table S1.** Clinico-pathologic characteristics of colon cancer cohorts

| **UCAN** |  |
| --- | --- |
| **Characteristic** | **Patients** |
|  | **N= 253** |
| **Age (Years)** |  |
| <66 | 73 (28.9%) |
| ≥66 | 180 (71.1%) |
| **Sex** |  |
| Female | 122 (48.2%) |
| Male | 131 (51.8%) |
| **Location** |  |
| Left | 119 (47.0%) |
| Right | 134 (53.0%) |
| **MMR status** |  |
| MSI | 50 (19.8%) |
| MSS | 196 (77.5%) |
| Missing data | 7 (2.8%) |
| **Stage** |  |
| I | 20 (7.9%) |
| II | 88 (34.8%) |
| III | 99 (39.1%) |
| IV | 42 (16.6) |
| Missing data | 4 (1.6%) |
| **Differentiation Grade** |  |
| High | 143 (56.5%) |
| Low | 45 (17.8%) |
| Missing data | 65 (25.7%) |
| **Adjuvant Chemotherapy** |  |
| No | 163 (64.4%) |
| Yes | 90 (35.6%) |

| **Nordic adjuvant randomized clinical trial** | |
| --- | --- |
| **Characteristic** | **Patients** |
|  | **N= 267** |
| **Age (Years)** |  |
| <66 | 111 (41.6%) |
| ≥66 | 156 (58.4%) |
| **Sex** |  |
| Female | 125 (46.8%) |
| Male | 142 (53.2%) |
| **Location** |  |
| Left | 117 (43.8%) |
| Right | 150 (56.2%) |
| **MMR status** |  |
| MSI | 44 (16.5%) |
| MSS | 204 (76.4%) |
| Missing data | 19 (7.1%) |
| **Stage** |  |
| II | 130 (48.7%) |
| III | 137 (51.3%) |
| **Differentiation Grade** |  |
| High | 198 (74.2%) |
| Low | 58 (21.7%) |
| Missing data | 11 (4.1%) |
| **Adjuvant Chemotherapy** |  |
| No | 137 (51.3%) |
| Yes | 130 (48.7%) |
