# Supplementary material for: Prognostic Interactions between FAP+ Fibroblasts and CD8a+ T Cells in Colon Cancer
Source: Cancers (Basel). 2020 Nov 3;12(11):3238. doi: 10.3390/cancers12113238 (PMC7693786; doi:10.3390/cancers12113238)
Supplement: Supplementary file 1 [file cancers-12-03238-s001.zip › cancers-854260-suppl.-final/Supp Tables/Table S2.docx]

**Table S2.** Clinico-pathological characteristics of patients in U-CAN cohort and their association with FAP intensity in the tumor center.

|  | **FAP intensity TC** | |  |
| --- | --- | --- | --- |
|  | number (percent) | |  |
| **Characteristic** | **Low** | **High** | **p-value** |
| **Age (Years)** |  |  |  |
| < 66 | 51 (69.9) | 22 (30.1) | 0.747 |
| ≥ 66 | 122 (67.8) | 58 (32.2) |  |
| **Sex** |  |  |  |
| Female | 85 (69.7) | 37 (30.3) | 0.670 |
| Male | 88 (67.2) | 43 (32.8) |  |
| **Location** |  |  |  |
| Left | 87 (73.1) | 32 (26.9) | 0.127 |
| Right | 86 (64.2) | 48 (35.8) |  |
| **Mismatch repair status** |  |  |  |
| MSI | 26 (52) | 24 (48) | 0.006** |
| MSS | 142 (72.4) | 54 (27.6) |  |
| **Stage** |  |  |  |
| I | 18 (90.0) | 2 (10.0) | 0.123 |
| II | 60 (68.2) | 28 (31.8) |  |
| III | 62 (62.6) | 37 (37.4) |  |
| IV | 29 (69) | 13 (31) |  |
| **Adjuvant Chemotherapy** |  |  |  |
| No | 118 (72.4) | 45 (27.4) | 0.065 |
| Yes | 55 (61.1) | 35 (38.9) |  |
| **CD8a density TC** |  |  |  |
| Low | 57(67.9) | 27(32.1) | 0.900 |
| High | 116(68.3) | 53(31.4) |  |

*< .05

**< .01

***< .001
