# Supplementary material for: Prognostic Interactions between FAP+ Fibroblasts and CD8a+ T Cells in Colon Cancer
Source: Cancers (Basel). 2020 Nov 3;12(11):3238. doi: 10.3390/cancers12113238 (PMC7693786; doi:10.3390/cancers12113238)
Supplement: Supplementary file 1 [file cancers-12-03238-s001.zip › cancers-854260-suppl.-final/Supp Tables/Table S3.docx]

|  | **CD8a density TC** | |  |
| --- | --- | --- | --- |
|  | number (percent) | |  |
| **Characteristic** | **Low** | **High** | **p-value** |
| **Age (Years)** |  |  |  |
| < 66 | 28 (38.4) | 45 (61.6) | 0.268 |
| ≥ 66 | 56 (31.1) | 124 (68.9) |  |
| **Sex** |  |  |  |
| Female | 33 (27) | 89 (73) | 0.045 |
| Male | 51 (38.9) | 80 (61.1) |  |
| **Location** |  |  |  |
| Left | 47 (39.5) | 72 (60.5) | 0.045 |
| Right | 37 (27.6) | 97 (72.4) |  |
| **Mismatch repair status** |  |  |  |
| MSI | 7 (14.0) | 43 (86.0) | 0.001** |
| MSS | 75 (38.3) | 121 (61.7) |  |
| **Stage** |  |  |  |
| I | 8 (40.0) | 12 (60.0) | 0.017* |
| II | 20 (22.7) | 68 (77.3) |  |
| III | 33 (33.3) | 66 (66.7) |  |
| IV | 21 (50.0) | 21 (50.0) |  |
| **Adjuvant Chemotherapy** |  |  |  |
| No | 50 (30.7) | 113 (69.3) | 0.251 |
| Yes | 34 (37.8) | 56 (62.2) |  |
| **FAP intensity TC** |  |  |  |
| Low | 57 (32.9) | 116 (67.1) | 0.900 |
| High | 27 (33.8) | 53 (66.3) |  |

**Table S3.** Clinico-pathological characteristics of the patients in the U-CAN cohort and their association with CD8a density in the tumor center.

*< .05

**< .01

***< .001
