# Supplementary material for: Prognostic Interactions between FAP+ Fibroblasts and CD8a+ T Cells in Colon Cancer
Source: Cancers (Basel). 2020 Nov 3;12(11):3238. doi: 10.3390/cancers12113238 (PMC7693786; doi:10.3390/cancers12113238)
Supplement: Supplementary file 1 [file cancers-12-03238-s001.zip › cancers-854260-suppl.-final/Supp Tables/Table S4.docx]

| *Non adjusted- Formal interaction test* | | |
| --- | --- | --- |
| *Covariates* | *HR (95%) CI* | *p-value* |
| FAP intensity TC (high vs low) | 1.062 (0.559-2.019) | 0.854 |
| CD8a density TC (high vs low) | 0.268 (0.119-0.605) | 0.002 |
| Combined FAP CD8a variable | 3.268 (1.287-8.304) | **0.013** |
| *Adjusted Formal- interaction test* | | |
| *Covariates* | *HR (95%) CI* | *p-value* |
| FAP intensity TC (high vs low) | 1.243 (0.636-2.430) | 0.525 |
| CD8a density TC (high vs low) | 0.218 (0.092-0.517) | 0.001 |
| Age (˃66 years or ≤ 66) | 1.204 (0.704-2.059) | 0.497 |
| Stage (III_IV vs I_II) | 7.160 (4.309-11.898) | 0.000 |
| MMR status (MSS vs MSI) | 0.972 (0.541-1.746) | 0.924 |
| Adjuvant treatment (Yes vs No) | 0.197 (0.114-0.341) | 0.000 |
| Location (Right or Left) | 1.160 (0.762-1.766) | 0.489 |
| Sex (Male or Female) | 0.705 (0.463-1.073) | 0.103 |
| Combined FAP CD8a variable | 4.463 (1.690-11.786) | **0.003** |

**Table S4**. Formal interaction test showing statistically significant interactions between FAP intensity and CD8a density markers in the prognostication of OS in the U-CAN cohort.
