# Supplementary material for: Prognostic Interactions between FAP+ Fibroblasts and CD8a+ T Cells in Colon Cancer
Source: Cancers (Basel). 2020 Nov 3;12(11):3238. doi: 10.3390/cancers12113238 (PMC7693786; doi:10.3390/cancers12113238)
Supplement: Supplementary file 1 [file cancers-12-03238-s001.zip › cancers-854260-suppl.-final/Supp Tables/Table S5.docx]

**Table S5.** **A)** Multivariable models of FAP intensity predicting survival of colon patients with low and high expression of CD8a in the U-CAN cohort. **B)** Multivariable models of CD8a density predicting survival of colon patients with low and high expression of FAP in the U-CAN cohort.

**A)**

|  | | | | | | | | |
| --- | --- | --- | --- | --- | --- | --- | --- | --- |
|  | **LOW CD8a density TC** | | | | **HIGH CD8a density TC** | | | |
| Covariates | HR | 95.0% CI for HR | | p-value | HR | 95.0% CI for HR | | p-value |
|  |  | Lower | Upper |  |  | Lower | Upper |  |
| **FAP intensity TC**  **(high vs low)** | 1.204 | 0.600 | 2.417 | 0.601 | 0.274 | 0.137 | 0.566 | **0.000***** |
| Age  (≥66 years or < 66) | 1.090 | 0.491 | 2.420 | 0.833 | 1.581 | 0.722 | 3.462 | 0.252 |
| Sex  (Male or Female) | 0.736 | 0.364 | 1.489 | 0.394 | 0.744 | 0.436 | 1.268 | 0.277 |
| Stage  (III_IV vs I_II) | 6.014 | 2.495 | 14.500 | **0.000***** | 7.760 | 4.116 | 14.630 | **0.000***** |
| Adjuvant treatment  (Yes vs No) | 0.143 | 0.059 | 0.344 | **0.000***** | 0.258 | 0.125 | 0.533 | **0.000***** |
| MMR status  (MSS vs MSI) | 0.923 | 0.251 | 3.395 | 0.941 | 0.907 | 0.466 | 1.766 | 0.774 |
| Location  (Right or Left) | 1.960 | 0.969 | 3.967 | 0.061 | 0.824 | 0.481 | 1.414 | 0.483 |

**B)**

|  | | | | | | | | |
| --- | --- | --- | --- | --- | --- | --- | --- | --- |
|  | **LOW FAP intensity TC** | | | | **HIGH FAP intensity TC** | | | |
| Covariates | HR | 95.0% CI for HR | | p-value | HR | 95.0% CI for HR | | p-value |
|  |  | Lower | Upper |  |  | Lower | Upper |  |
| **CD8a density TC**  **(high vs low)** | 0.929 | 0.563 | 1.534 | 0.774 | 0.165 | 0.050 | 0.541 | **0.003**** |
| Age  (≥66 years or < 66) | 1.395 | 0.753 | 2.587 | 0.290 | 0.984 | 0.304 | 3.189 | 0.979 |
| Sex  (Male or Female) | 0.784 | 0.488 | 1.260 | 0.314 | 0.440 | 0.166 | 1.166 | 0.099 |
| Stage  (III_IV vs I_II) | 5.360 | 3.106 | 9.250 | **0.000***** | 35.253 | 6.619 | 187.761 | **0.000***** |
| Adjuvant treatment  (Yes vs No) | 0.239 | 0.126 | 0.454 | **0.000***** | 0.098 | 0.033 | 0.293 | **0.000***** |
| MMR status  (MSS vs MSI) | 1.000 | 0.510 | 1.958 | 0.999 | 0.852 | 0.244 | 2.978 | 0.802 |
| Location  (Right or Left) | 1.153 | 0.714 | 1.860 | 0.561 | 1.053 | 0.408 | 2.718 | 0.915 |

*< .05; **< .01; ***< .001
