# Supplementary material for: Prognostic Interactions between FAP+ Fibroblasts and CD8a+ T Cells in Colon Cancer
Source: Cancers (Basel). 2020 Nov 3;12(11):3238. doi: 10.3390/cancers12113238 (PMC7693786; doi:10.3390/cancers12113238)
Supplement: Supplementary file 1 [file cancers-12-03238-s001.zip › cancers-854260-suppl.-final/Supp Tables/Table S6.docx]

**Table S6**. Formal interaction test showing statistically significant interactions between FAP intensity and CD8a density markers in the prognostication of OS in subsets of patients in the U-CAN cohort.

| *Non adjusted- Formal interaction test* | | | |
| --- | --- | --- | --- |
| *Combined FAP CD8a variable* | | *HR (95%) CI* | *p-value* |
| Age | ˃66 years | 0.101 (0.009-1.084) | 0.058 |
|  | ≤ 66 years | 0.440 (0.152-1.270) | 0.129 |
| Gender | Female | 0.711 (0.176-2.872) | 0.632 |
|  | Male | 0.135 (0.032-0.568) | 0.006 |
| Location | Left | 0.484 (0.120-1.950) | 0.308 |
|  | Right | 0.248 (0.069-0.896) | 0.033 |
| MRR status | MSI | * |  |
|  | MSS | 0.258 (0.082-0.807) | 0.020 |
| Adjuvant Treatment | No | 0.324 (0.104-1.010) | 0.052 |
|  | Yes | 0.170 (0.030-0.964) | 0.045 |
| Stage | I_II | 0.374 (0.021-6.682) | 0.503 |
|  | III_IV | 0.312 (0.112-0.868) | 0.026 |

*Interaction test was not performed since one of the groups lacked events.
