# Supplementary material for: Prognostic Interactions between FAP+ Fibroblasts and CD8a+ T Cells in Colon Cancer
Source: Cancers (Basel). 2020 Nov 3;12(11):3238. doi: 10.3390/cancers12113238 (PMC7693786; doi:10.3390/cancers12113238)
Supplement: Supplementary file 1 [file cancers-12-03238-s001.zip › cancers-854260-suppl.-final/Supp Tables/Table S7.docx]

|  | **FAP intensity TC** | |  | **CD8a density TC** | |  |
| --- | --- | --- | --- | --- | --- | --- |
|  | number (percent) | |  | number (percent) | |  |
| **Characteristic** | **low** | **high** | **p-value** | **low** | **high** | **p-value** |
| **Age (Years)** |  |  |  |  |  |  |
| < 66 | 44 (39.6) | 67 (60.4) | 0.820 | 19 (17.1) | 92 (82.9) | 0.757 |
| ≥ 66 | 64 (41.0) | 92 (59.0) |  | 29 (18.6) | 127 (81.4) |  |
| **Sex** |  |  |  |  |  |  |
| Female | 55 (44.0) | 70 (56.0) | 0.269 | 22 (17.6) | 103 (82.4) | 0.880 |
| Male | 53 (37.3) | 89 (62.7) |  | 26 (18.3) | 116 (81.7) |  |
| **Location** |  |  |  |  |  |  |
| Left | 44 (37.6) | 73 (62.4) | 0.403 | 22 (18.8) | 95 (81.2) | 0.756 |
| Right | 64 (42.7) | 86 (57.3) |  | 26 (17.3) | 124 (82.7) |  |
| **Mismatch repair status** |  |  |  |  |  |  |
| MSI | 14 (31.8) | 30 (68.2) | 0.205 | 4 (9.19) | 40 (90.9) | 0.075 |
| MSS | 86 (42.2) | 118 (57.8) |  | 42 (20.6) | 162 (79.4) |  |
| **Stage** |  |  |  |  |  |  |
| II | 58 (44.6) | 72 (55.4) | 0.177 | 27 (20.8) | 103 (79.2) | 0.247 |
| III | 50 (36.5) | 87 (63.5) |  | 21 (15.3) | 116 (84.7) |  |
| **Adjuvant Chemotherapy** |  |  |  |  |  |  |
| No | 55 (40.1) | 82 (59.9) | 0.917 | 28 (20.4) | 109 (79.6) | 0.282 |
| Yes | 53 (40.8) | 77 (59.2) |  | 20 (15.4) | 110 (84.6) |  |
| **Differentiation Grade** |  |  |  |  |  |  |
| High | 82 (41.4) | 116 (58.6) | 0.477 | 41 (20.7) | 157 (79.3) | 0.002** |
| Low | 21 (36.2) | 37 (63.8) |  | 2 (3.4) | 56 (96.6) |  |
|  |  |  |  |  |  |  |

**Table S7.** Clinico-pathological characteristics of patients in the “Nordic adjuvant randomized clinical trial” and their association with FAP intensity and CD8a density in the tumor center.

*< .05

**< .01

***< .001
