# Supplementary material for: Prognostic Interactions between FAP+ Fibroblasts and CD8a+ T Cells in Colon Cancer
Source: Cancers (Basel). 2020 Nov 3;12(11):3238. doi: 10.3390/cancers12113238 (PMC7693786; doi:10.3390/cancers12113238)
Supplement: Supplementary file 1 [file cancers-12-03238-s001.zip › cancers-854260-suppl.-final/Supp Tables/Table S8.docx]

| *Non adjusted- Formal interaction test* | | |
| --- | --- | --- |
| *Covariates* | *HR (95%) CI* | *p-value* |
| FAP intensity TC (high vs low) | 1.633 (0.720-3.704) | 0.240 |
| CD8a density TC (high vs low) | 0.504 (0.263-0.968) | 0.039 |
| Combined FAP CD8a variable | 2.512 (1.008-6.263) | **0.048** |
| *Adjusted- Formal interaction test* | | |
| *Covariates* | *HR (95%) CI* | *p-value* |
| FAP intensity TC (high vs low) | 1.353 (0.514-3.556) | 0.540 |
| CD8a density TC (high vs low) | 0.585 (0.260-1.319) | 0.196 |
| Age (˃66 years or ≤ 66) | 1.129 (0.853-1.950) | 0.227 |
| Stage (III_IV vs I_II) | 2.260 (1.489-3.431) | 0.000 |
| MMR status (MSS vs MSI) | 2.354 (1.212-4.572) | 0.011 |
| Adjuvant treatment (Yes vs No) | 0.960 (0.647-1.426) | 0.840 |
| Location (Right or Left) | 1.243 (0.821-1.883) | 0.303 |
| Sex (Male or Female) | 1.280 (0.852-1.923) | 0.235 |
| Differentiation (low or high) | 1.247 (0.754-2.062) | 0.390 |
| Combined FAP CD8a variable | 2.472 (0.859-7.112) | 0.093 |

**Table S8**. Formal interaction test showing statistically significant interactions between FAP intensity and CD8a density markers in the prognostication of OS in the “Nordic adjuvant randomized clinical trial”.
